# Temporal changes in *Plasmodium falciparum* genetic diversity and multiplicity of infection across three areas of varying malaria transmission intensities in Uganda

**DOI:** 10.1186/s41182-024-00672-7

**Published:** 2024-12-30

**Authors:** Alex Mwesigwa, Steven M. Kiwuwa, Benson Musinguzi, Hakiim Kawalya, James Davis Katumba, Andrew Baguma, Irene M. Mutuku, Ismail Abiola Adebayo, Samuel L. Nsobya, Pauline Byakika-Kibwika, Joan N. Kalyango, Charles Karamagi, Joaniter I. Nankabirwa

**Affiliations:** 1 Clinical Epidemiology Unit, School of Medicine, Makerere University College of Health Sciences, P. O. Box 7072, Kampala, Uganda; 2 Department of Microbiology and Immunology, School of Medicine, Kabale University, P. O. Box 314, Kabale, Uganda; 3 Department of Biochemistry, School of Biomedical Sciences, College of Health Sciences, Makerere, University, P.O. Box 7072, Kampala, Uganda; 4 Departent of Medical Laboratory Science, Faculty of Health Sciences, Muni University, P.O. Box 725, Arua, Uganda; 5 Department of Immunology and Molecular Biology, Makerere University College of Health Sciences, P. O. Box 7072, Kampala, Uganda; 6 Health Tutors’ College, Mulago, P.O. Box 5225, Kampala, Uganda; 7 Infectious Diseases Research Collaboration (IDRC), P.O. Box 7475, Kampala, Uganda; 8 Mbarara University of Science and Technology, Mbarara, Uganda

**Keywords:** *P. falciparum*, Genetic diversity, Multiplicity of infection, Transmission intensity

## Abstract

**Background:**

Malaria is a significant public health challenge in Uganda, with *Plasmodium falciparum* (*P. falciparum*) responsible for most of malaria infections. The high genetic diversity and multiplicity of infection (MOI) associated with *P. falciparum* complicate treatment and prevention efforts. This study investigated temporal changes in *P. falciparum* genetic diversity and MOI across three sites with varying malaria transmission intensities. Understanding these changes is essential for informing effective malaria control strategies for the different malaria transmission settings.

**Methods:**

A total of 220 *P. falciparum*-positive dried blood spot (DBS) filter paper samples from participants in a study conducted during 2011–2012 and 2015–2016 were analyzed. Genotyping utilized seven polymorphic markers: Poly-α, TA1, TA109, PfPK2, 2490, C2M34–313, and C3M69–383. Genetic diversity metrics, including the number of alleles and expected heterozygosity, were calculated using GENALEX and ARLEQUIN software. MOI was assessed by counting distinct genotypes. Multi-locus linkage disequilibrium (LD) and genetic differentiation were evaluated using the standardized index of association (I_A_^S^) and Wright's fixation index (F_ST_), respectively. Statistical comparisons were made using the Kruskal–Wallis test, and temporal trends were analyzed using the Jonckheere–Terpstra test, with statistical significance set at *p* < 0.05.

**Results:**

Of the 220 samples, 180 were successfully amplified. The majority of participants were males (50.6%) and children aged 5–11 years (46.7%). Genetic diversity remained high, with mean expected heterozygosity (H_e_) showing a slight decrease over time (range: 0.73–0.82). Polyclonal infections exceeded 50% at all sites, and mean MOI ranged from 1.7 to 2.2, with a significant reduction in Tororo (from 2.2 to 2.0, *p* = 0.03). Linkage disequilibrium showed a slight increase, with Kanungu exhibiting the lowest I_A_^S^ in 2011–2012 (0.0085) and Jinja the highest (0.0239) in 2015–2016. Overall genetic differentiation remained low, with slight increases in pairwise F_ST_ values over time, notably between Jinja and Tororo (from 0.0145 to 0.0353).

**Conclusions:**

This study highlights the genetic diversity and MOI of *P. falciparum* in Uganda's malaria transmission settings, noting a slight decrease in both genetic diversity and MOI overtime. Continued surveillance and targeted control strategies are essential for monitoring the impact of malaria control efforts in Uganda.

**Supplementary Information:**

The online version contains supplementary material available at 10.1186/s41182-024-00672-7.

## Background

Although the malaria burden in Uganda steadily declined in the last two decades [1], malaria remains a significant public health concern [2]. According to the World Health Organization (WHO) Malaria Report 2023, there were 249 million malaria cases recorded globally, with 233 million occurring in sub-Saharan Africa (SSA), contributing to an estimated 580,000 out of 608,000 malaria-related deaths worldwide in 2022 alone. Uganda accounted for 5% of global malaria cases and ranked as the third-highest contributor to malaria cases [3]. More than 90% of these malaria cases are caused by *Plasmodium falciparum* [4], which poses high morbidity and mortality compared to other species [5].

Malaria control is hindered by several factors, including the high genetic diversity of *P. falciparum* parasites, the frequent occurrence of multiplicity of infection (MOI). MOI, which is the presence of multiple genetic variants or genotypes within a single infection [6], complicates efforts to control the disease. It usually occurs in two ways: when an individual is bitten by different mosquitoes carrying unique parasite strains (superinfection), or when a single mosquito transmits multiple distinct genotypes in a single bite (co-transmission) [7, 8]. The genetic diversity of *P. falciparum* arises primarily from genetic recombination during the parasite’s lifecycle in the mosquito [9]. Increased genetic recombination leads to low linkage disequilibrium (LD) within parasite populations, meaning that alleles at different loci become more randomly associated. As a result, it becomes less likely for specific genetic variants to be inherited together [10–12].

The presence of diverse and multiple *P. falciparum* strains within an individual enhances parasite virulence and contributes to the pathology of malaria [13, 14]. Parasite diversity also plays a significant role in the development of drug resistance [15], posing major challenges to malaria control and elimination efforts, such as the use of artemisinin-based combination therapies (ACTs) and long-lasting insecticide-treated bed nets (LLINs) [16]. Furthermore, the extensive genetic variability of *P. falciparum* vaccine targets complicates the development of an effective vaccine [17, 18]. Therefore, understanding *P. falciparum* genetic diversity and MOI at the population level, as well as the dynamics of this diversity, is crucial for informing malaria control strategies [19]. In addition, longitudinal analysis of LD offers valuable insights into the temporal changes in the genetic structure of *P. falciparum* parasite populations [20]. Advanced techniques such as whole genome sequencing (WGS) and targeted deep sequencing are sensitive methods for assessing parasite genetic diversity. However, these methods remain costly and inaccessible in many SSA regions [21]. In contrast, microsatellite markers, including Poly-α, TA1, TA109, and PfPK2, offer cost-effective and unbiased alternatives for evaluating *P. falciparum* genetic diversity [10, 11, 22]. These markers, which are neutral polymorphic loci abundant in the *P. falciparum* genome, consist of repeat motifs like [TA]n, [T]n, and [TAA]n [23]. Due to their high variability, they allow for differentiation of parasite strains and the assessment of MOI across various genomic regions.

Previous studies have shown that the genetic diversity of *P. falciparum* varies across individuals, populations, transmission settings, as well as with fluctuations in parasite prevalence [24–27]. This variability makes *P. falciparum* genetic diversity and MOI effective tools for tracking changes in malaria transmission intensity [28–30], and assessing the impact of interventions on malaria transmission patterns [31–33]. In areas with intense malaria transmission, such as Kenya [34] and Senegal [35], high genetic diversity and the presence of multiple parasite genotypes are consistently observed. In these regions, *P. falciparum* parasites are characterized by weak LD, high genetic diversity, and minimal population differentiation, which are key indicators of high malaria transmission intensity [10]. Conversely, parasites circulating in low malaria transmission areas, such as São Tomé, exhibit reduced genetic diversity, strong LD, and significant population differentiation [36]. While these trends reflect the influence of malaria transmission intensity on the genetic structure of *P. falciparum* populations, similar patterns have not always been observed in other low transmission regions [37]. In addition, *P. falciparum* MOI tends to decrease as malaria transmission intensity declines over time [38]. These patterns are particularly relevant for Uganda, where malaria transmission intensity varies considerably across regions with some areas experiencing high transmission, while others have low transmission [39]. Understanding the dynamics of *P. falciparum* genetic diversity and MOI within Uganda is critical for effective malaria control and elimination strategies.

Despite the importance of genetic diversity and MOI as essential tools for monitoring malaria transmission, there is limited data on the temporal changes in *P. falciparum* genetic diversity and MOI in Uganda, particularly in relation to fluctuating transmission intensities. Most studies have primarily focused on malaria case numbers from high transmission areas [40–42], which may not offer a comprehensive understanding of malaria transmission dynamics over time. In addition, research on *P. falciparum* genetic diversity and MOI in Uganda has mostly been cross-sectional, examining single timepoints [14, 22, 43]. Consequently, there is a gap in knowledge regarding how these factors evolve over time within the country. This study addressed these gaps by investigating the dynamics of *P. falciparum* genetic diversity and MOI using isolates from participants enrolled at three sites with varying transmission intensities, over two distinct time periods: 2011–2012 and 2015–2016. The aim was to better understand how transmission intensity influences parasite genetic diversity and MOI in Uganda.

## Methods

### Study settings

**Fig. 1:**
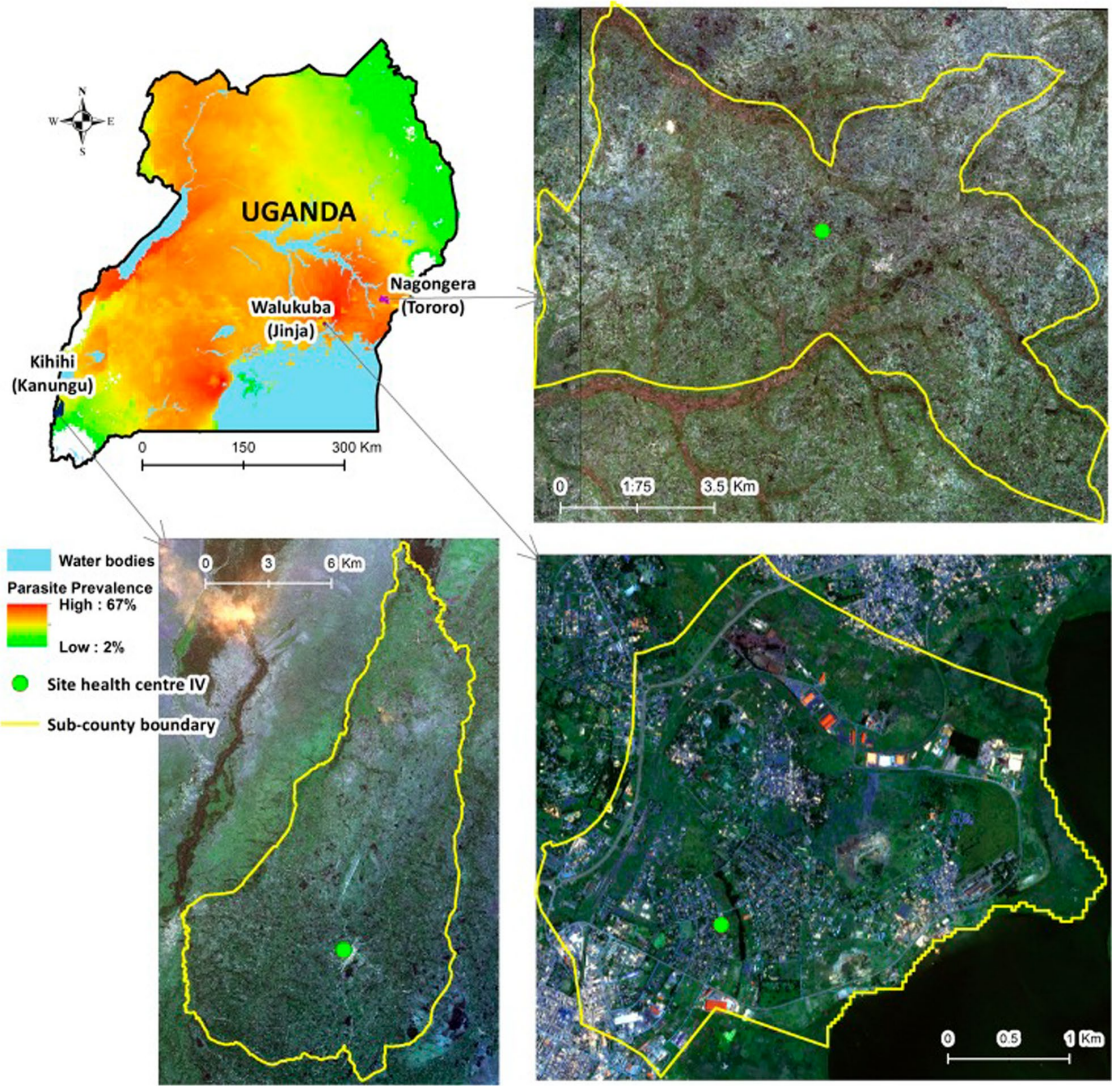
Map of Uganda showing the malaria endemicity of the study sites at the time of sample collection. Adapted from Kamya et al. [39]

This study utilized dried blood spot (DBS) samples collected from participants enrolled in the Program for Resistance, Immunology, Surveillance, and Modeling of Malaria (PRISM) study at two distinct timepoints: 2011–2012 and 2015–2016. Participants were recruited from three sub-counties with varying malaria transmission intensities, determined by the number of malaria cases: Walukuba in Jinja District, Kihihi in Kanungu District, and Nagongera in Tororo District. Walukuba is a relatively low-transmission, peri-urban area near Lake Victoria in the south-central region of Uganda. Kihihi, a rural area near Bwindi Impenetrable National Park in the southwest, has moderate transmission intensity. In contrast, Nagongera, a rural area near the border with Kenya in the southeast, experiences high transmission intensity (Fig. 1). A total of 220 DBS samples were retrieved, with the distribution across study sites and time periods as follows: 40 samples from Walukuba (Jinja District), 35 from Kihihi (Kanungu District), and 33 from Nagongera (Tororo District) during the 2011-2012 study period. In the 2015–2016 period, 35 samples were collected from Walukuba, 39 from Kihihi, and 38 from Nagongera.

### Study population

Participants were enrolled in the PRISM study at two distinct timepoints: from August 2011 to December 2012 and from July 2015 to December 2016. Detailed information about the study population has been described previously [39]. Briefly, all households in the three sub-counties were enumerated and mapped, and 100 households were randomly selected for participation. Children aged 0.5–10 years, along with one primary adult caregiver from each household, were enrolled. This participant recruitment approach was designed to provide a comprehensive understanding of factors influencing malaria infection and transmission patterns across different age groups within households. Participants were encouraged to visit a clinic open 7 days a week for medical care. Routine visits were conducted every 3 months, during which standardized evaluations were performed. Blood samples were collected by finger prick for thick blood smears, hemoglobin measurements, and DBS filter paper samples for future molecular studies. At each visit, participants with a fever (tympanic temperature > 38.0 °C) or a history of fever within the previous 24 h had a thick blood smear read immediately. If the smear was positive, the patient was diagnosed with malaria and treated according to national guidelines.

### Laboratory methods

Laboratory assays, including malaria microscopy and parasite genotyping, were conducted at the Molecular Biology Research Laboratory at the Infectious Diseases Research Collaboration (IDRC) in Kamapala-Uganda.

### Determination of parasite density

Microscopy slides for parasite density and species detection were prepared using a 10% Giemsa solution and stained for 30 minutes, with both thick and thin blood films being prepared. Experienced microscopists examined the slides under a light microscope at 100 × oil immersion. *P. falciparum* parasite density was determined by counting asexual parasites against 200 leukocytes. The parasite density per µL of blood was calculated by multiplying the total parasite count by 40, assuming an average of 8,000 leukocytes per µL of blood [44]. For quality control, each smear was independently read by two microscopists. Though discrepancies—defined as differences in species diagnosis, parasite density > 50%, or presence of parasites—were rare, any identified discrepancy prompted a review by a third microscopist. To minimize discrepancies, all microscopists underwent thorough training on standardized techniques and parasite identification prior to the study, and regular assessments were conducted to ensure consistent performance. Final parasitemia was determined by averaging the readings of the two microscopists or, in cases of disagreement, by averaging the third microscopist's reading with that of the closest of the initial two. In cases where the third microscopist’s reading was significantly different, it was used as the final determination for parasite density and or species.

### Selection of the samples for molecular analysis

A stratified random sampling approach was employed to ensure that the molecular analysis included participants from diverse demographic and clinical groups, reflecting the broader study population. A total of 220 DBS filter paper samples were selected from two study periods: 2011–2012 and 2015–2016. The selection criteria included availability of DBS filter paper sample, *P. falciparum* mono-infection positivity, and sufficient demographic and clinical data for each participant. To achieve a representative sample, the selection was stratified based on key factors, including age, gender, geographical location, and study period. The selected DBS filter paper samples were then linked to participants' demographic and clinical information via cohort ID, as well as the date and year of sample collection.

### DNA extraction

Genomic DNA of *P. falciparum* was extracted from dried blood spots (DBS) using Chelex 100 Resin (Sigma-Aldrich, USA), following the method described by Musapa et al.[45]. The Chelex extraction method was chosen for its simplicity, cost-effectiveness, and efficiency in processing DBS samples. It effectively isolates *P. falciparum* DNA, reduces PCR inhibitors, and requires fewer reagents, offering a practical and reliable alternative to silica-based or column-based methods. Briefly, 6 mm discs were punched out from the DBS into 1.5 mL microcentrifuge tubes containing 1 mL of 1X phosphate-buffered saline (PBS) and incubated overnight at 4 °C. The punching machine was cleaned with DNase, and a clean blank piece of Whatman 3MM filter paper was pre-cut between samples to prevent cross-contamination. The discs were washed twice with 1 mL PBS and then boiled at 99 °C in 200 μL of 20% Chelex (Sigma-Aldrich, USA) in DNase/RNase-free water. After a final centrifugation step (14,000 × g for 1 min), the extracted DNA was transferred into a labelled 0.6 mL microcentrifuge tube with a 100 µL elution volume and then stored at − 20 °C until further use. Detection and confirmation of *P. falciparum* was performed through genotyping of *P. falciparum* 18S rRNA using nested PCR [46]. As an internal control, every eighth sample consisted of a blank filter paper sample that was cut, extracted, and processed alongside the field samples to identify any contamination that could lead to false positives.

### Microsatellite genotyping

A panel of seven neutral polymorphic microsatellites of *P. falciparum* was genotyped, including TA1 (Chr6), Poly-α (Chr4), PfPK2 (Chr12), TA109 (Chr6), 2490 (Chr10), C2M34–313 (Chr2), and C3M69–383 (Chr3). Primers labeled with HEX or 6-FAM were used for genotyping at the Infectious Diseases Research Collaboration (IDRC) Molecular Biology Laboratory in Kampala, Uganda (Additional file 1, Table S1). The microsatellites Poly-α, TA1, TA109, PfPK2, and 2490 were nested, while C2M34–313 and C3M69–383 were unnested.

For the nested PCR reactions, the primary reaction for each marker was carried out in a total volume of 15 µL, containing 10.5 µL of molecular-grade PCR water, 1.5 µL of 10 × reaction buffer, 0.3 µL of dNTPs (1.25 mM), 0.3 µL of Forward Primer (10 μM), 0.3 µL of Reverse Primer (10 μM), 0.25 µL of AmpliTaq Gold (5 U/µL), and 2 µL of DNA template. The Round 1 PCR conditions were as follows: 94 °C for 2 min, followed by 25 cycles of (94 °C for 30 s, 42 °C for 30 s, 40 °C for 30 s, and 65 °C for 40 s), and ending with 65 °C for 2 min. The secondary reaction contained the same reagents as the primary reaction, with the addition of 0.3 µL of the labeled primer for each marker. A 2 µL aliquot of the primary reaction product was used in a final volume of 15 µL for the nested PCR reactions. The Round 2 PCR conditions were: 94 °C for 2 min, followed by 25 cycles of (94 °C for 20 s, 45 °C for 20 s, and 65 °C for 30 s), and ending with 65 °C for 2 min.

PCR conditions for the C2M34–313 and C3M69–383 reactions were as follows: 94 °C for 2 min, followed by 5 cycles of (94 °C for 30 s, 50 °C for 30 s, and 60 °C for 30 s), then 40 cycles of (94 °C for 30 s, 45 °C for 30 s, and 60 °C for 30 s), and ending with 60 °C for 2 min. A 2 µL sample of the PCR product was then run on a 2% agarose gel to confirm amplification before being analyzed on the sequencer. The amplified PCR products were transferred to safe-lock DNA amplicon storage tubes, securely wrapped in aluminum foil, and sent to Inqaba Biotec in South Africa for microsatellite fragment analysis using an ABI capillary electrophoresis platform.

### Microsatellite analysis

Microsatellite fluorescent-labeled PCR products were analyzed using an Applied Biosystems ABI 3730xl Genetic Analyzer (Thermo Fisher Scientific, Waltham, MA, USA) to determine their length. The peaks were scored using GeneMarker HID V2.9.5 software. For samples that produced more than one peak, the highest peak was defined as the dominant allele. Additional peaks were classified as minor alleles if their peak heights exceeded 200 relative fluorescence units (RFU) and were > 20% of the height of the dominant peak. This threshold was used to identify minor alleles, which may represent clones present at lower frequencies but still contribute to the genetic diversity of the infection. The identification of these additional minor alleles, including third and fourth alleles, was based on the relative peak heights at each microsatellite locus. Peaks that met these criteria were recorded as distinct alleles.

### *P. falciparum* genetic diversity

The genetic diversity of *P. falciparum*, usually resulting from genetic recombination [47], was assessed in each parasite population from each study site by calculating the mean number of alleles (N_a_), and the number of effective alleles (N_e_) across each locus. These metrics were calculated from the predominant allele data set using GENALEX 6.5 software [48]. Expected heterozygosity (H_e_), defined as the probability that two randomly selected clones from a population will carry distinct alleles at each marker locus, was calculated using ARLEQUIN software version 3.11 [49] with the formula:

$${\text{H}}_{{\text{e}}} \, = \,\left[ {{\text{n}}/\left( {{\text{n }} - { 1}} \right)} \right]\,\left[ {{1 } - \Sigma {\text{p}}_{{\text{i}}}^{{2}} } \right],$$

where ‘*n*’ represents the number of isolates analyzed and ‘*pi*’ is the frequency of the i^th^ allele in a given population. H_e_ values range between 0 (no genetic diversity) and 1 (high genetic diversity) [10]. The mean N_a_, N_e_, and H_e_ values for each study site were computed as the mean of the values from each locus.

### *P. falciparum* MOI

*P. falciparum* MOI was defined as the number of distinct parasite genotypes co-existing within a given infection [50]. Isolates with only one allele were considered monoclonal infections, while those with more than one allele were classified as polyclonal infections [51]. The MOI for each infection was determined by identifying the highest number of alleles observed across any of the microsatellite markers used in the analysis. This maximum allele count was considered the MOI for that particular infection. To assess the parentage of polyclonal infections, we counted the number of isolates with more than one allele for each microsatellite marker that successfully amplified. The results were then summarized across the different study sites and time periods.

### Analysis of multi-locus linkage disequilibrium and genetic differentiation

Multi-locus linkage disequilibrium (LD) measured as the standardized index of association (I_A_^S^) was calculated using the program LIAN version 3.5 [52] for the whole data set. This index was calculated using the formula:

$${\text{I}}_{{\text{A}}}^{{\text{S}}} \, - \, = \,\left( {{1}/{\text{n}} - {1}\left( {{\text{VD}}/\left( {{\text{VE}}} \right)\, - \,1} \right)} \right)$$

where VE is the expected variance of the n^th^ number of loci for which two individuals differ. VD is the observed variance. The significance of the I_A_^S^ values was tested using the Monte Carlo method. Genetic diferentiation was assessed using Wrights fixation index (F_ST_) calculated using ARLEQUIN software version 3.11 [49]. The F_ST_ values ranging from 0 to 0.05 indicates low genetic variability, 0.05–0.15 indicates moderate genetic variability, 0.15–0.25 indicated high great genetic differentiation and > 0.25 indicates substantial genetic differentiation [53].

### Data analysis

Participants’ demographic and clinical data, including age, gender, parasite density, and hemoglobin levels, were extracted from the primary PRISM cohort database and exported to STATA version 17 (Stata Corp., College Station, TX, USA) for analysis. These data were summarized using descriptive statistics, such as means and proportions. Microsatellite data were retrieved from the ABI 3730xl Genetic Analyzer. Genetic analysis was performed only on samples, where at least five microsatellite markers were successfully amplified. To minimize bias associated with multiple infections, only the predominant alleles were included in the analysis. Samples with incomplete or poor-quality amplification, or failure to amplify on at least 3 markers, were excluded from further analysis to ensure the accuracy and reliability of the data. Statistical comparisons of *P. falciparum* genetic diversity (including the mean N_a_, N_e_ and H_e_) and MOI, as measured by mean MOI and the percentage of polyclonal infections, were performed using Kruskal–Wallis test. Temporal trends in these indices were assessed using the Jonckheere–Terpstra test. Statistical significance was set at *p* < 0.05.

## Results

### Characteristics of the study population

Of the 220 *P. falciparum* positive samples selected, 180 (81.8%) successfully amplified on at least five microsatellites and were included in the final analysis. Of the 180 samples, 91 (50.6%) were from male participants, and many (46.7%) were from participants aged 5–11 years of age. Overall, the mean parasite densities in the study areas decreased over time. In the 2011–2012 study period, the mean parasite counts were 17,034 parasites/µL in Walukuba Subcounty (Jinja District), 36,118 parasites/µL in Kihihi Subcounty (Kanungu District), and 68,240 parasites/µL in Nongongera Subcounty (Tororo District) (Table 1). By the 2015–2016 period, these counts had declined to 14,882.4 parasites/µL in Walukuba, 20,694 parasites/µL in Kihihi, and 23,586.2 parasites/µL in Nongongera (Table 1). Regarding hemoglobin (Hb) levels among study participants, Walukuba Subcounty experienced an increase from 11.3 to 11.7 g/dL, and Nongongera Subcounty rose from 11.1 to 11.3 g/dL. In contrast, Kihihi Subcounty noted a decrease in mean Hb levels from 10.9 to 10.7 g/dL during the same period (Table 1).

**Table 1:** Demographic characteristics of the study participants whose samples are included in the analysis

Characteristic	Isolates collected 2011–2012			Isolates collected 2015–2016			Overall
	Jinja (*n* = 38)	Kanungu (*n* = 27)	Tororo (*n* = 24)	Jinja (*n* = 26)	Kanungu (*n* = 34)	Tororo (*n* = 31)	
Age in years							
< 5 years	21	14	14	8	12	11	80
5–11 years	11	13	9	15	22	14	84
≥ 18 years	6	0	1	3	0	6	16
Gender (%)							
Male	52.6	55.6	58.3	50	47.1	41.9	50.9
Mean Axillary temperature, ^0^c (SD)	38.6(0.9)	38.4(1.8)	38.5(0.8)	36.8(0.5)	38.3(1.2)	37.3(0.9)	37(1.2)
Mean Hb g/dL(SD)	11.3(1.9)	10.9(2)	11.1(1.5)	11.7(1.7)	10.7(2.4)	11.3(2.3)	11.1(2.1)
Mean parasite density/µL (SD)	17,034 (2,736.1)	36,118 (4,157.9)	68,240 (8,491)	14,882.4 (1,181)	20,694 (2,197.1)	23,586.2 (1,927)	30,092 (2,750)

### Temporal changes in *P. falciparum* genetic diversity between 2011–2012 and 2015–2016 study periods across sites

**Fig. 2:**
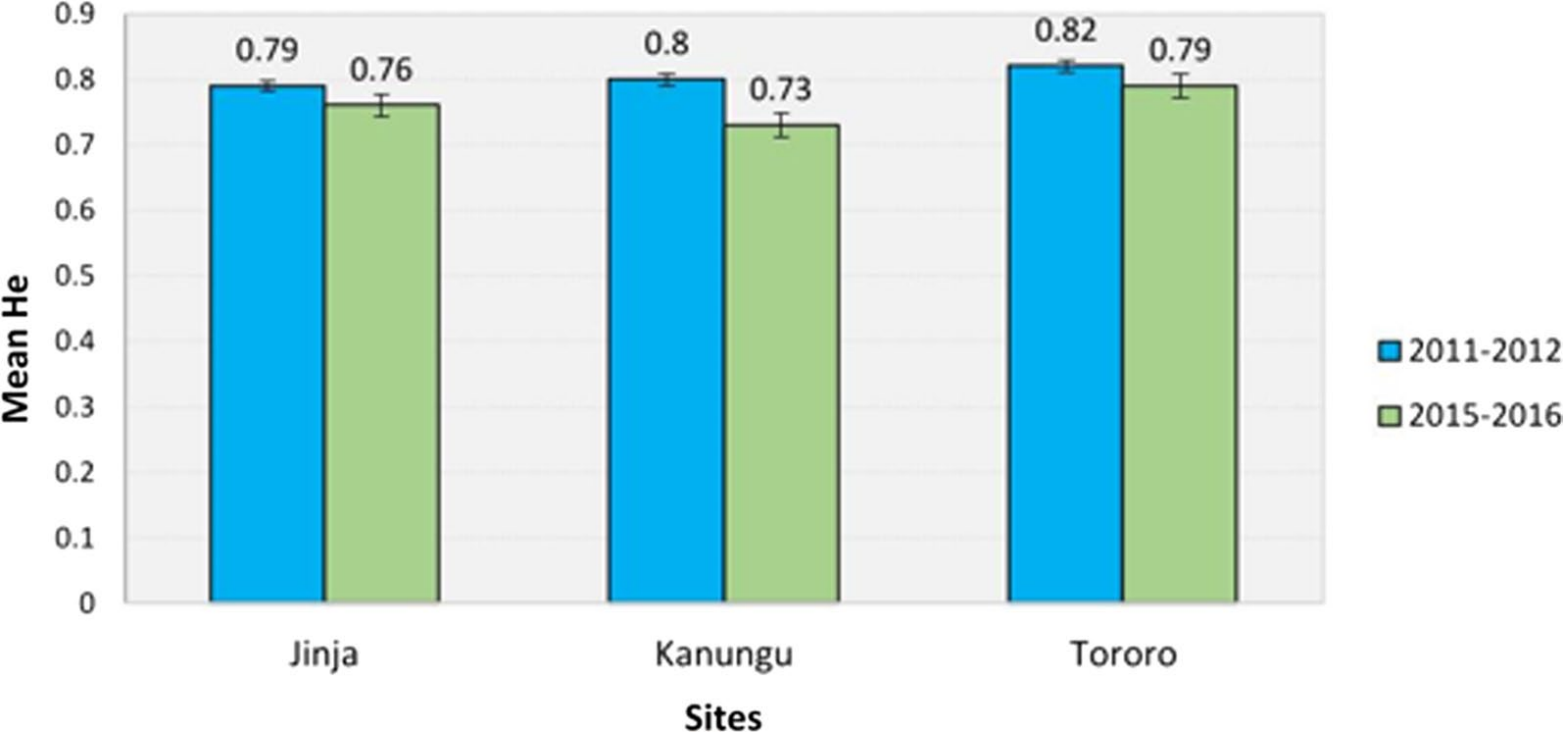
Changes in *P. falciparum* mean H_e_ values between 2011–2012 and 2015–2016 study periods across sites

Overall, *P. falciparum* genetic diversity remained consistently high across all study sites over time, with mean H_e_ values consistently exceeding 0.7 (Fig. 2). However, a slight decline in the mean values of N_a_, N_e_, and H_e_ was observed across all study sites. Importantly, no significant differences were observed in mean N_a_, N_e_, or H_e_ between the study periods, nor were there any significant temporal trends in these indices across the two study periods. These findings were supported by the non-significant results from both the Kruskal–Wallis and Jonckheere–Terpstra tests (*p* > 0.05) (Fig. 2; Additional file 2, Table S2).

### Temporal changes in *P. falciparum* MOI between the 2011–2012 and 2015–2016 study periods across sites

**Fig. 3:**
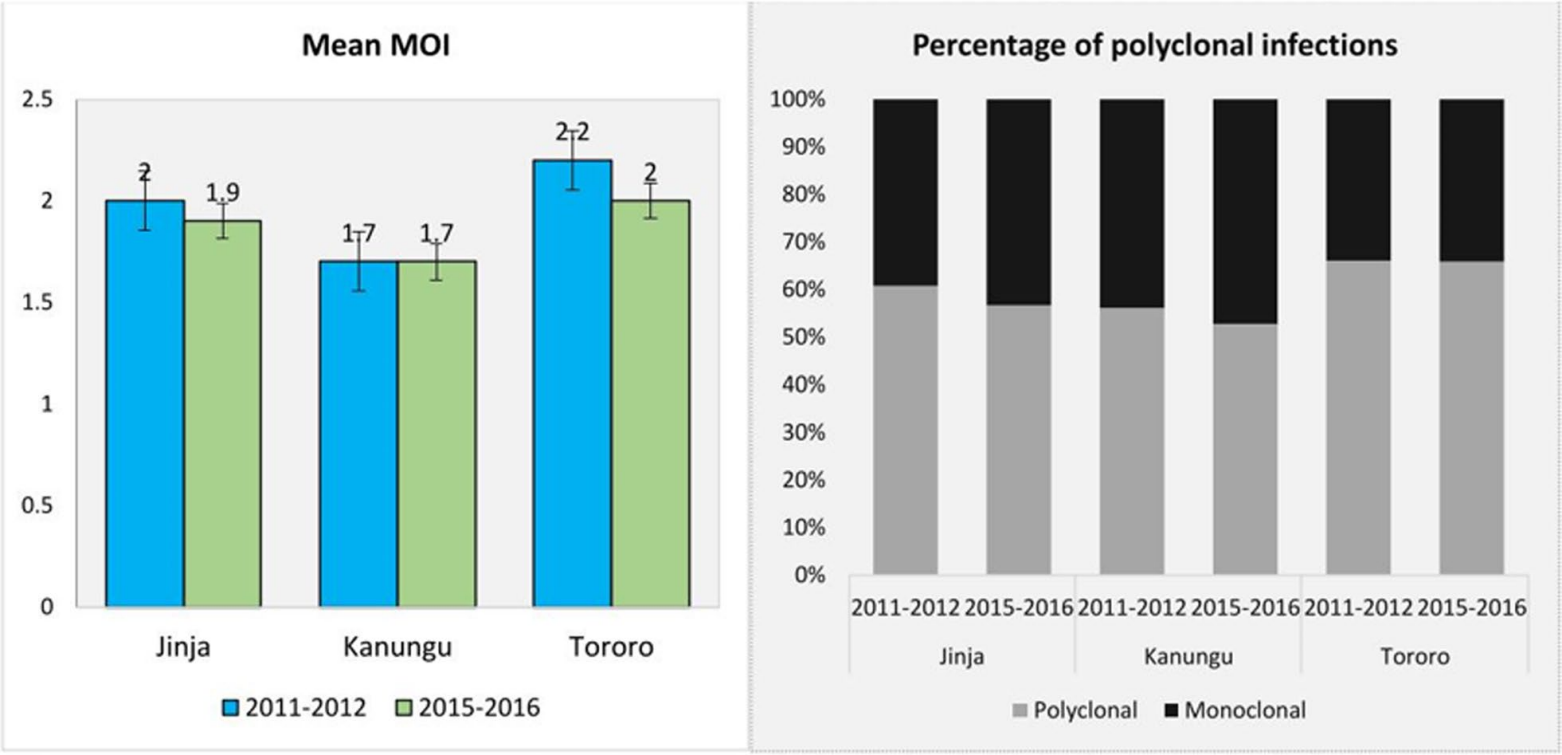
Changes in *P. falciparum* mean MOI and the percentage of polyclonal infections between 2011–2012 and 2015–2016 study periods across sites

Overall, the percentage of *P. falciparum* polyclonal infections in the three study areas remained consistently high, exceeding 50%, which indicates a robust polyclonal parasite population over time. During the study periods, there was a significant temporal decrease in the percentage of polyclonal infections in Kanungu, declining from 56.1 to 52.8% overtime (*p* = 0.01). In contrast, Jinja and Tororo showed no significant temporal differences in the percentage of polyclonal infections (*p* > 0.05). Regarding *P. falciparum* mean MOI, there was a significant temporal reduction in Tororo, where it decreased from 2.2 to 2.0 overtime (*p* = 0.03) (Fig. 3; Additional file 3, Table S3). However, Jinja and Kanungu did not exhibit significant changes in mean MOI over time (*p* > 0.05) (Fig. 3).

### Temporal changes in *P. falciparum* population multi-locus linkage disequilibrium and genetic differentiation between the 2011–2012 and 2015–2016 study periods across study sites

#### Temporal changes in linkage disequilibrium (LD) among *P. falciparum* populations

A multilocus index of association analysis was performed to assess the non-random associations of all microsatellite loci in the data set. The statistical significance of LD was tested using 10,000 Monte Carlo simulations. Overall, there was a slight increase in LD (based on the standardized index of association (I_A_^S^)) across the study sites over time. During the 2011–2012 period, Kanungu recorded the lowest I_A_^S^ at 0.0085, while Jinja had the highest significant I_A_^S^ value at 0.0233 (*p* = 0.01). In the 2015–2016 study period, Kanungu's I_A_^S^ rose to 0.0114, and in Tororo it increased to 0.0168. Jinja also experienced a slight increase in I_A_^S^, from 0.0233 to 0.0239 (Table 2).

**Table 2:** Changes in *P. falciparum* parasites’ linkage disequilibrium between the 2011–2012 and 2015–2016 study periods across study sites

Site/population	2011–2012		2015–2016	
	I_A_^S^	*P* value	I_A_^S^	*P* value
Jinja	0.0233	0.06	0.0239	0.01
Kanungu	0.0085	0.31	0.0114	0.14
Tororo	0.0097	0.26	0.0168	0.18

#### Temporal changes in genetic differentiation of *P. falciparum* populations

Overall, the genetic differentiation of the *P. falciparum* population among the study sites was low, with a slight increase between the study periods. In the 2011–2012 period, the F_ST_ values were low: 0.0275 between Jinja and Kanungu, and 0.0145 between Jinja and Tororo, with the highest differentiation at 0.0503 between Kanungu and Tororo. In the 2015–2016 study period, F_ST_ values showed modest increases: 0.0384 between Jinja and Kanungu, and 0.0353 between Jinja and Tororo. The highest differentiation remained between Kanungu and Tororo, increasing to 0.0585 (Table 3).

**Table 3:** Changes in pairwise genetic differentiation (F_ST_) between the 2011–2012 and 2015–2016 study periods among study sites

2011–2012				2015–2016			
	Jinja	Kanungu	Tororo		Jinja	Kanungu	Tororo
Jinja	0.0000	0.0275	0.0145	Jinja	0.0000	0.0384	0.0353
Kanungu	0.0275	0.0000	0.0503	Kanungu	0.0384	0.0000	0.0585
Tororo	0.0145	0.0503	0.0000	Tororo	0.0353	0.0585	0.0000

## Discussion

*P. falciparum* genetic diversity and MOI are influenced by several factors, including malaria transmission intensity and the effectiveness of control interventions [31, 54]. To our knowledge, no studies have assessed temporal changes in *P. falciparum* genetic diversity and MOI across different malaria transmission areas in Uganda. This study examined temporal changes in *P. falciparum *genetic diversity and MOI of malaria parasites in regions with varying transmission intensities. The findings revealed slight decreases in both genetic diversity and MOI between 2011–2012 and 2015–2016. This suggests a relatively stable maintenance of high parasite genetic diversity and MOI across the three study areas, underscoring the importance of continued surveillance and monitoring of malaria control efforts.

Overall, the genetic diversity of *P. falciparum* remained generally high, with mean expected heterozygosity (H_e_) values exceeding 0.7 across all study areas over time. This likely reflects significant malaria transmission intensity, even in regions like Jinja, which is classified as having low transmission. These findings align with reports from other regions, including Eswatini, where malaria transmission has decreased due to intensive control measures (e.g., insecticide-treated nets, indoor residual spraying, and access to diagnostics and treatment), yet *P. falciparum* genetic diversity remains high due to the importation of malaria parasites from neighboring areas with high transmission [55, 56]. This highlights that even in areas with reduced malaria transmission, regional transmission dynamics and ongoing malaria parasites importation continue to maintain high levels of genetic diversity. Similarly, in Kenya, regions with high malaria transmission intensity still exhibit high *P. falciparum* genetic diversity (mean H_e_ ~ 0.78), despite substantial control interventions, such as insecticide-treated nets, indoor residual spraying [34], and the use of artemisinin-based combination therapies [57], which have been implemented. The persistence of high genetic diversity in both low- and high-malaria transmission settings suggests that gene flow, driven by human migration and cross-border transmission, plays a key role in sustaining genetic diversity. This further supports the idea that malaria transmission intensity is not the sole determinant of *P. falciparum* genetic diversity.

Our findings suggest that *P. falciparum* populations possess substantial capacity to adapt to environmental pressures, such as antimalarial drugs and insecticides [21, 58, 59]. The high genetic diversity, along with low genetic differentiation between study sites, implies that these populations form interconnected reproductive units, likely shaped by ongoing gene flow [60]. This is consistent with a recent study by Arinaitwe et al*.* [61] which suggested that human mobility is a driver of malaria transmission and the maintenance of large parasite reservoirs in Uganda. Our study also underscores the complexity of inferring malaria transmission intensity from genetic data alone. External factors like human migration and malaria importation from neighboring high-transmission areas complicate the relationship between genetic diversity and local transmission. This is evident in regions like Eswatini and Kenya, where local transmission and regional dynamics influence genetic diversity, demonstrating the importance of considering broader transmission networks when evaluating malaria transmission intensity.

Previous studies have indicated that high *P. falciparum* MOI values are commonly found in regions with intense malaria transmission [62, 63] and are directly linked to transmission intensity [64]. Our study also observed a slight temporal decrease in MOI, with values in Jinja and Tororo dropping from 2.0 to 1.9 and from 2.2 to 2.0, respectively, in line with a gradual decline in transmission intensity in these areas (Table 1). These decreases align with findings from Tororo [65], which indicated reduced transmission following the implementation of vector control measures. Research from Grande Comore Island also demonstrated a decrease in *P. falciparum* mean MOI values—declining from 3.11 to 1.63 for *msp-1* and from 2.75 to 1.35 for *msp-2*—after the introduction of artemisinin-based combination therapy (ACT) [66]. These trends contrast with studies from the Democratic Republic of the Congo (DRC), where MOI increased from 3.78 in to 4.64 [67], and in Kenya, where MOI increased from 1.7 to 3.0 [68] despite ongoing interventions. This suggests that the relationship between transmission intensity and MOI is not uniform and can vary by region and intervention effectiveness. Moreover, our study found that polyclonal infections, constituting more than 50% of infections across the three study areas, were common, in line with observations from mesoendemic and holoendemic regions [68, 69]. However, some studies have reported a decrease in polyclonal infections following control interventions, suggesting that control measures may also influence the genetic structure of the parasite population [66].

The longitudinal analysis of LD provided additional insights into the genetic structure of *P. falciparum* populations. In regions with high transmission intensity, where multiple genetically diverse *P. falciparum* strains circulate, low LD typically reflects ongoing genetic recombination between these diverse strains during the sexual phase of the parasite’s lifecycle [70]. Conversely, in areas with lower transmission intensity, lower LD can sometimes reflect the persistence of clonal populations of *P. falciparum*, especially in cases of clonal expansion following a bottleneck [71]. Our study revealed a slight increase in LD between 2011–2012 and 2015–2016, which may reflect reduced genetic recombination, potentially due to a decrease in malaria transmission intensity. This finding aligns with previous studies that noted increased LD in areas with reduced malaria transmission [51]. The significantly high LD values observed in Jinja during 2011–2012 are suggestive of a clonal population structure, which is typical of low-transmission areas [26, 72]. In contrast, study sites like Kanungu and Tororo exhibited lower LD levels, reflecting the high transmission intensity in these regions.

As malaria transmission dynamics evolve, continuous monitoring of parasite genetic diversity and MOI is crucial for adapting control strategies. Our study offers several strengths. It includes diverse sampling locations with varying malaria transmission intensities, providing a comprehensive comparison of genetic diversity and MOI across different transmission settings. The longitudinal design, spanning 2011–2016, tracks changes in parasite genetics over time, offering valuable insights into the impact of malaria control interventions. In addition, the use of microsatellite genotyping provides detailed insights into *P. falciparum* genetic diversity and population dynamics.

However, there are some limitations to our study. The reliance on neutral microsatellite markers may not fully capture the complexity of *P. falciparum* populations, particularly in the context of evolving control strategies. While microsatellites remain a cost-effective and accessible tool for assessing genetic diversity and MOI, especially in regions like Uganda, where advanced genomic technologies are less accessible, they have certain limitations. Furthermore, challenges such as sample amplification issues, unequal sample sizes across sites and timepoints, and potential selection bias due to participant mobility may have influenced our results. To address these limitations, we recommend future studies to incorporate newer technologies, such as whole-genome sequencing (WGS) or deep amplicon sequencing, and expand recruitment across multiple locations to better capture transmission dynamics and minimize potential biases.

## Conclusion

This study provides valuable insights into *P. falciparum* genetic diversity and MOI across Uganda’s diverse malaria transmission settings. Despite slight decreases in both genetic diversity and MOI between the 2011–2012 and 2015–2016 study periods, overall genetic diversity remained high, reflecting the parasite’s robust transmission dynamics and ability to adapt to environmental pressures. The findings highlight the need for continued surveillance and adaptive malaria control strategies, ensuring that interventions remain effective across regions with varying transmission intensities. By considering regional transmission dynamics, human mobility, and the impact of control measures, future strategies can better target malaria control efforts and address the evolving challenges of malaria management in Uganda.

## Supplementary Information

Supplementary material 1.

Supplementary material 2.

Supplementary material 3.

Supplementary material 4.

## Data Availability

No datasets were generated or analysed during the current study.

## Abbreviations

ACT: Artemisinin based combination therapy
DBS: Dried blood spot
DRC: Democratic Republic of Congo
Hb: Hemoglobin
H_e_: Expected heterozygosity
IDRC: Infectious diseases research collaboration
LD: Linkage disequilibrium
LLIN: Long-lasting insecticide-treated bed nets
MOI: Multiplicity of infection
MSP: Merozoite surface protein
N_a_: Number of alleles
N_e_: Number of effective alleles
PBS: Phosphate-buffered saline
PCR: Polymerase chain reaction
PRISM: Program for resistance, immunology and modeling of malaria
RFU: Relative fluorescence units
SSA: Sub-Saharan Africa
WGS: Whole genome sequencing

## References

[1] Kigozi SP, Kigozi RN, Sebuguzi CM, Cano J, Rutazaana D, Opigo J, et al. Spatial-temporal patterns of malaria incidence in Uganda using HMIS data from 2015 to 2019. BMC Public Health. 2020;20(1):1–14. 33317487 10.1186/s12889-020-10007-w PMC7737387

[2] Nankabirwa JI, Briggs J, Rek J, Arinaitwe E, Nayebare P, Katrak S, et al. Persistent parasitemia despite dramatic reduction in malaria incidence after 3 rounds of indoor residual spraying in Tororo. Uganda J Infect Dis. 2019;219(7):1104–11. 30383230 10.1093/infdis/jiy628 PMC6420168

[3] WHO. World malaria report 2023. Geneva: World Health Organization; 2023.

[4] Asua V, Tukwasibwe S, Conrad M, Walakira A, Nankabirwa JI, Mugenyi L, et al. Plasmodium species infecting children presenting with malaria in Uganda. Am J Trop Med Hyg. 2017;97(3):753–7. 28990911 10.4269/ajtmh.17-0345 PMC5590612

[5] WHO. World malaria report 2019. Geneva: World Health Organisation; 2019.

[6] Pinkevych M, Petravic J, Bereczky S, Rooth I, Färnert A, Davenport MP. Understanding the relationship between *Plasmodium falciparum* growth rate and multiplicity of infection. J Infect Dis. 2015;211(7):1121–7. 25301957 10.1093/infdis/jiu561

[7] Alizon S. Parasite co-transmission and the evolutionary epidemiology of virulence. Evol. 2013;67(4):921–33. 10.1111/j.1558-5646.2012.01827.x 23550745

[8] Wong W, Griggs AD, Daniels RF, Schaffner SF, Ndiaye D, Bei AK, et al. Genetic relatedness analysis reveals the cotransmission of genetically related *Plasmodium falciparum* parasites in Thiès, Senegal. Genome Med. 2017;9(1):5. 28118860 10.1186/s13073-017-0398-0 PMC5260019

[9] Mu J, Awadalla P, Duan J, McGee KM, Joy DA, McVean GAT, et al. Recombination hotspots and population structure in *Plasmodium falciparum*. PLoS Biol. 2005;3(10):e335. 16144426 10.1371/journal.pbio.0030335 PMC1201364

[10] Anderson TJ, Haubold B, Williams JT, Estrada-Franco JG, Richardson L, Mollinedo R, et al. Microsatellite markers reveal a spectrum of population structures in the malaria parasite *Plasmodium falciparum*. Mol Biol Evol. 2000;17(10):1467–82. 11018154 10.1093/oxfordjournals.molbev.a026247

[11] Carter TE, Malloy H, Existe A, Memnon G, St. Victor Y, Okech BA, et al. Genetic diversity of *Plasmodium falciparum* in Haiti: insights from microsatellite markers. PLoS One. 2015;10(10):e0140416. 26462203 10.1371/journal.pone.0140416 PMC4604141

[12] Su X, Hayton K, Wellems TE. Genetic linkage and association analyses for trait mapping in *Plasmodium falciparum*. Nat Rev Genet. 2007;8(7):497–506. 17572690 10.1038/nrg2126

[13] Jensen AR, Adams Y, Hviid L. Cerebral Plasmodium falciparum malaria: the role of PfEMP1 in its pathogenesis and immunity, and PfEMP1-based vaccines to prevent it. Immunol Rev. 2020;293(1):230–52. 31562653 10.1111/imr.12807 PMC6972667

[14] Kiwuwa MS, Ribacke U, Moll K, Byarugaba J, Lundblom K, Färnert A, et al. Genetic diversity of *Plasmodium falciparum* infections in mild and severe malaria of children from Kampala, Uganda. Parasitol Res. 2013;112(4):1691–700. 23408340 10.1007/s00436-013-3325-3 PMC3597336

[15] Narh CA, Ghansah A, Duffy MF, Ruybal-Pesántez S, Onwona CO, Oduro AR, et al. Evolution of antimalarial drug resistance markers in the reservoir of *Plasmodium falciparum* infections in the Upper East Region of Ghana. J Infect Dis. 2020;222(10):1692–701. 32459360 10.1093/infdis/jiaa286 PMC7982568

[16] Ghosh SK, Ghosh C. New challenges in malaria elimination. In: Rodriguez-Morales AJ, editor. Current topics and emerging issues in malaria elimination. London: Intechopen; 2021. p. 133.

[17] Healer J, Murphy V, Hodder AN, Masciantonio R, Gemmill AW, Anders RF, et al. Allelic polymorphisms in apical membrane antigen-1 are responsible for evasion of antibody-mediated inhibition in *Plasmodium falciparum*. Mol Microbiol. 2004;52(1):159–68. 15049818 10.1111/j.1365-2958.2003.03974.x

[18] Genton B, Betuela I, Felger I, Al-Yaman F, Anders RF, Saul A. A recombinant blood-stage malaria vaccine reduces Plasmodium falciparum density and exerts selective pressure on parasite populations in a phase 1–2b trial in Papua New Guinea. J Infect Dis. 2002;185(6):820–7. 11920300 10.1086/339342

[19] Gwarinda HB, Tessema SK, Raman J, Greenhouse B, Birkholtz L-M. Parasite genetic diversity reflects continued residual malaria transmission in Vhembe District, a hotspot in the Limpopo Province of South Africa. Malar J. 2021;20:1–13. 33593382 10.1186/s12936-021-03635-z PMC7885214

[20] Chenet SM, Taylor JE, Blair S, Zuluaga L, Escalante AA. Longitudinal analysis of Plasmodium falciparum genetic variation in Turbo, Colombia: implications for malaria control and elimination. Malar J. 2015;14:363. 10.1186/s12936-015-0887-9 PMC4578328 26395166

[21] Apinjoh TO, Ouattara A, Titanji VP, Djimde A, Amambua-Ngwa A. Genetic diversity and drug resistance surveillance of Plasmodium falciparum for malaria elimination: is there an ideal tool for resource-limited sub-Saharan Africa? Malar J. 2019;18(1):217. 31242921 10.1186/s12936-019-2844-5 PMC6595576

[22] Agaba BB, Anderson K, Gresty K, Prosser C, Smith D, Nankabirwa JI, et al. Genetic diversity and genetic relatedness in *Plasmodium falciparum* parasite population in individuals with uncomplicated malaria based on microsatellite typing in Eastern and Western regions of Uganda, 2019–2020. Malar J. 2021;20(1):242. 34059047 10.1186/s12936-021-03763-6 PMC8165787

[23] Su X-z, Wellems TE. Toward a high-resolution *Plasmodium falciparum* Linkage map: polymorphic markers from hundreds of simple sequence repeats. Genomics. 1996;33(3):430–44. 8661002 10.1006/geno.1996.0218

[24] Abukari Z, Okonu R, Nyarko SB, Lo AC, Dieng CC, Salifu SP, et al. The diversity, multiplicity of infection and population structure of *P*. *falciparum* parasites circulating in asymptomatic carriers living in high and low malaria transmission settings of Ghana. Genes. 2019;10(6):434. 31181699 10.3390/genes10060434 PMC6628376

[25] Adjah J, Fiadzoe B, Ayanful-Torgby R, Amoah LE. Seasonal variations in *Plasmodium falciparum* genetic diversity and multiplicity of infection in asymptomatic children living in southern Ghana. BMC Infect Dis. 2018;18:1–10. 30157794 10.1186/s12879-018-3350-z PMC6114730

[26] Pumpaibool T, Arnathau C, Durand P, Kanchanakhan N, Siripoon N, Suegorn A, et al. Genetic diversity and population structure of Plasmodium falciparum in Thailand, a low transmission country. Malar J. 2009;8:1–11. 19602241 10.1186/1475-2875-8-155 PMC2722663

[27] Childs LM, Prosper OF. Simulating within-vector generation of the malaria parasite diversity. PLoS ONE. 2017;12(5):e0177941. 28542484 10.1371/journal.pone.0177941 PMC5440164

[28] Neafsey DE, Volkman SK. Malaria genomics in the era of eradication. Cold Spring Harb Perspect Med. 2017;7(8):a025544. 28389516 10.1101/cshperspect.a025544 PMC5538406

[29] Pringle JC, Tessema S, Wesolowski A, Chen A, Murphy M, Carpi G, et al. Genetic evidence of focal *Plasmodium falciparum* transmission in a pre-elimination setting in Southern Province, Zambia. J Infect Dis. 2019;219(8):1254–63. 30445612 10.1093/infdis/jiy640 PMC6452320

[30] Auburn S, Barry AE. Dissecting malaria biology and epidemiology using population genetics and genomics. Int J Parasitol. 2017;47(2–3):77–85. 27825828 10.1016/j.ijpara.2016.08.006

[31] Daniels R, Chang H-H, Séne PD, Park DC, Neafsey DE, Schaffner SF, et al. Genetic surveillance detects both clonal and epidemic transmission of malaria following enhanced intervention in Senegal. PLoS ONE. 2013;8(4):e60780. 23593309 10.1371/journal.pone.0060780 PMC3617153

[32] Daniels RF, Schaffner SF, Wenger EA, Proctor JL, Chang H-H, Wong W, et al. Modeling malaria genomics reveals transmission decline and rebound in Senegal. Proc Natl Acad Sci U S A. 2015;112(22):7067–72. 25941365 10.1073/pnas.1505691112 PMC4460456

[33] Escalante AA, Ferreira MU, Vinetz JM, Volkman SK, Cui L, Gamboa D, et al. Malaria molecular epidemiology: lessons from the International Centers of Excellence for Malaria Research Network. Am J Trop Med Hyg. 2015;93(3 Suppl):79–86. 26259945 10.4269/ajtmh.15-0005 PMC4574277

[34] Gatei W, Gimnig JE, Hawley W, Ter Kuile F, Odero C, Iriemenam NC, et al. Genetic diversity of *Plasmodium falciparum* parasite by microsatellite markers after scale-up of insecticide-treated bed nets in western Kenya. Malar J. 2015;14:1–12. 26651480 10.1186/s12936-015-1003-x PMC4675068

[35] Niang M, Thiam LG, Loucoubar C, Sow A, Sadio BD, Diallo M, et al. Spatio-temporal analysis of the genetic diversity and complexity of *Plasmodium falciparum* infections in Kedougou, southeastern Senegal. Parasit Vectors. 2017;10(1):33. 28103905 10.1186/s13071-017-1976-0 PMC5244544

[36] Chen Y-A, Shiu T-J, Tseng L-F, Cheng C-F, Shih W-L, de Assunção Carvalho AV, et al. Dynamic changes in genetic diversity, drug resistance mutations, and treatment outcomes of falciparum malaria from the low-transmission to the pre-elimination phase on the islands of São Tomé and Príncipe. Malar J. 2021;20:1–15. 34906134 10.1186/s12936-021-04007-3 PMC8672503

[37] Branch OH, Sutton PL, Barnes C, Castro JC, Hussin J, Awadalla P, et al. *Plasmodium falciparum* genetic diversity maintained and amplified over 5 years of a low transmission endemic in the Peruvian Amazon. Mol Biol Evol. 2011;28(7):1973–86. 21109587 10.1093/molbev/msq311 PMC3112368

[38] Niang M, Thiam LG, Loucoubar C, Sow A, Sadio BD, Diallo M, et al. Spatio-temporal analysis of the genetic diversity and complexity of *Plasmodium falciparum* infections in Kedougou, southeastern Senegal. Parasit Vectors. 2017;10(1):1–9. 28103905 10.1186/s13071-017-1976-0 PMC5244544

[39] Kamya MR, Arinaitwe E, Wanzira H, Katureebe A, Barusya C, Kigozi SP, et al. Malaria transmission, infection, and disease at three sites with varied transmission intensity in Uganda: implications for malaria control. Am J Trop Med Hyg. 2015;92(5):903–12. 25778501 10.4269/ajtmh.14-0312 PMC4426576

[40] Kigozi SP, Kigozi RN, Sebuguzi CM, Cano J, Rutazaana D, Opigo J, et al. Spatial-temporal patterns of malaria incidence in Uganda using HMIS data from 2015 to 2019. BMC Public Health. 2020;20:1–14. 33317487 10.1186/s12889-020-10007-w PMC7737387

[41] Nankabirwa JI, Arinaitwe E, Rek J, Kilama M, Kizza T, Staedke SG, et al. Malaria transmission, infection, and disease following sustained indoor residual spraying of insecticide in Tororo, Uganda. Am J Trop Med Hyg. 2020;103(4):1525. 32700666 10.4269/ajtmh.20-0250 PMC7543828

[42] Siya A, Kalule BJ, Ssentongo B, Lukwa AT, Egeru A. Malaria patterns across altitudinal zones of Mount Elgon following intensified control and prevention programs in Uganda. BMC Infect Dis. 2020;20:1–16. 10.1186/s12879-020-05158-5 PMC7301530 32552870

[43] Kyabayinze DJ, Karamagi C, Kiggundu M, Kamya MR, Wabwire-Mangen F, Kironde F, et al. Multiplicity of *Plasmodium falciparum* infection predicts antimalarial treatment outcome in Ugandan children. Afri Health Sc. 2008;8(4):200–5. PMC2887011 20589125

[44] WHO. Basic malaria microscopy. Geneva: World Health Organization; 2010.

[45] Musapa M, Kumwenda T, Mkulama M, Chishimba S, Norris DE, Thuma PE, et al. A simple Chelex protocol for DNA extraction from Anopheles spp. J Vis Exp. 2013;71:3281. 10.3791/3281 PMC3658367 23328684

[46] Singh B, Bobogare A, Cox-Singh J, Snounou G, Abdullah MS, Rahman HA. A genus-and species-specific nested polymerase chain reaction malaria detection assay for epidemiologic studies. Am J Trop Med Hyg. 1999;60(4):687–92. 10348249 10.4269/ajtmh.1999.60.687

[47] Miles A, Iqbal Z, Vauterin P, Pearson R, Campino S, Theron M, et al. Indels, structural variation, and recombination drive genomic diversity in *Plasmodium falciparum*. Genome Res. 2016;26(9):1288–99. 27531718 10.1101/gr.203711.115 PMC5052046

[48] Peakall R, Smouse PE. GENALEX 6: genetic analysis in Excel. Population genetic software for teaching and research. Bioinformatics. 2012;28(19):2537–9. 22820204 10.1093/bioinformatics/bts460 PMC3463245

[49] Excoffier L, Laval G, Schneider S. Arlequin (version 3.0): an integrated software package for population genetics data analysis. Evol Bioinform Online. 2007;1:47–50. 19325852 PMC2658868

[50] Sondo P, Derra K, Rouamba T, Nakanabo Diallo S, Taconet P, Kazienga A, et al. Determinants of *Plasmodium falciparum* multiplicity of infection and genetic diversity in Burkina Faso. Parasit Vectors. 2020;13:1–12. 32819420 10.1186/s13071-020-04302-z PMC7441709

[51] Mohd Abd Razak MR, Sastu UR, Norahmad NA, Abdul-Karim A, Muhammad A, Muniandy PK, et al. Genetic diversity of *Plasmodium falciparum* populations in malaria declining areas of Sabah, East Malaysia. PloS ONE. 2016;11(3):e0152415. 27023787 10.1371/journal.pone.0152415 PMC4811561

[52] Haubold B, Hudson RR. LIAN 3.0: detecting linkage disequilibrium in multilocus data. Bioinformatics. 2000;16(9):847–9. 11108709 10.1093/bioinformatics/16.9.847

[53] Balloux F, Lugon-Moulin N. The estimation of population differentiation with microsatellite markers. Mol Ecol. 2002;11(2):155–65. 11856418 10.1046/j.0962-1083.2001.01436.x

[54] Karl S, White MT, Milne GJ, Gurarie D, Hay SI, Barry AE, et al. Spatial effects on the multiplicity of *Plasmodium falciparum* infections. PLoS ONE. 2016;11(10):e0164054. 27711149 10.1371/journal.pone.0164054 PMC5053403

[55] Roh ME, Tessema SK, Murphy M, Nhlabathi N, Mkhonta N, Vilakati S, et al. High genetic diversity of *Plasmodium falciparum* in the low-transmission setting of the kingdom of Eswatini. J Infect Dis. 2019;220(8):1346–54. 31190073 10.1093/infdis/jiz305 PMC6743842

[56] Nkya TE, Fillinger U, Dlamini M, Sangoro OP, Marubu R, Zulu Z, et al. Malaria in Eswatini, 2012–2019: a case study of the elimination effort. Malar J. 2021;20:1–13. 33743727 10.1186/s12936-021-03699-x PMC7980328

[57] Musuva A, Ejersa W, Kiptui R, Memusi D, Abwao E. The malaria testing and treatment landscape in Kenya: results from a nationally representative survey among the public and private sector in 2016. Malar J. 2017;16:1–13. 29268789 10.1186/s12936-017-2089-0 PMC5740898

[58] Lefevre T, Ohm J, Dabiré KR, Cohuet A, Choisy M, Thomas MB, et al. Transmission traits of malaria parasites within the mosquito: genetic variation, phenotypic plasticity, and consequences for control. Evol Appl. 2017;11(4):456–69. 29636799 10.1111/eva.12571 PMC5891056

[59] Vardo-Zalik AM, Zhou G, Zhong D, Afrane YA, Githeko AK, Yan G. Alterations in *Plasmodium falciparum* genetic structure two years after increased malaria control efforts in western Kenya. Am J Trop Med Hyg. 2013;88(1):29. 23166196 10.4269/ajtmh.2012.12-0308 PMC3541741

[60] Ajogbasile FV, Kayode AT, Oluniyi PE, Akano KO, Uwanibe JN, Adegboyega BB, et al. Genetic diversity and population structure of *Plasmodium falciparum* in Nigeria: insights from microsatellite loci analysis. Malar J. 2021;20(1):236. 34039364 10.1186/s12936-021-03734-x PMC8152046

[61] Arinaitwe E, Dorsey G, Nankabirwa JI, Kigozi SP, Katureebe A, Kakande E, et al. Association between recent overnight travel and risk of malaria: a prospective cohort study at 3 sites in Uganda. Clin Infect Dis. 2019;68(2):313–20. 29868722 10.1093/cid/ciy478 PMC6321857

[62] Ghanchi NK, Mårtensson A, Ursing J, Jafri S, Bereczky S, Hussain R, et al. Genetic diversity among *Plasmodium falciparum* field isolates in Pakistan measured with PCR genotyping of the merozoite surface protein 1 and 2. Malar J. 2010;9:1–6. 20043863 10.1186/1475-2875-9-1 PMC2806377

[63] Akoniyon OP, Akiibinu M, Adeleke MA, Maharaj R, Okpeku M. A comparative study of genetic diversity and multiplicity of infection in uncomplicated *Plasmodium falciparum* infections in selected regions of pre-elimination and high transmission settings using MSP1 and MSP2 genes. Pathogens. 2024;13(2):172. 38392910 10.3390/pathogens13020172 PMC10891941

[64] Agyeman-Budu A, Brown C, Adjei G, Adams M, Dosoo D, Dery D, et al. Trends in multiplicity of *Plasmodium falciparum* infections among asymptomatic residents in the middle belt of Ghana. Malar J. 2013;12:1–6. 23327681 10.1186/1475-2875-12-22 PMC3558338

[65] Oguttu DW, Matovu JK, Okumu DC, Ario AR, Okullo AE, Opigo J, et al. Rapid reduction of malaria following introduction of vector control interventions in Tororo District, Uganda: a descriptive study. Malar J. 2017;16:1–8. 28558701 10.1186/s12936-017-1871-3 PMC5450094

[66] Huang B, Tuo F, Liang Y, Wu W, Wu G, Huang S, et al. Temporal changes in genetic diversity of *msp-1, msp-2*, and *msp-3* in *Plasmodium falciparum* isolates from Grande Comore Island after introduction of ACT. Malar J. 2018;17:1–14. 29458365 10.1186/s12936-018-2227-3 PMC5819244

[67] Pringle JC, Wesolowski A, Berube S, Kobayashi T, Gebhardt ME, Mulenga M, et al. High *Plasmodium falciparum* genetic diversity and temporal stability despite control efforts in high transmission settings along the international border between Zambia and the Democratic Republic of the Congo. Malar J. 2019;18:1–13. 31801548 10.1186/s12936-019-3023-4 PMC6894251

[68] Kimenyi KM, Wamae K, Ngoi JM, de Laurent ZR, Ndwiga L, Osoti V, et al. Maintenance of high temporal *Plasmodium falciparum* genetic diversity and complexity of infection in asymptomatic and symptomatic infections in Kilifi, Kenya from 2007 to 2018. Malar J. 2022;21(1):192. 35725456 10.1186/s12936-022-04213-7 PMC9207840

[69] Yakubu B, Longdet IY, Horsefield T, Davou DT, Obishakin E. High-complexity *Plasmodium falciparum* infections, north Central Nigeria, 2015–2018. Emerg Infect Dis. 2019;25(7):1330–8. 31211682 10.3201/eid2507.181614 PMC6590735

[70] Mulenge FM, Hunja CW, Magiri E, Culleton R, Kaneko A, Aman RA. Genetic diversity and population structure of *Plasmodium falciparum* in Lake Victoria Islands, a region of intense transmission. Am J Trop Med Hyg. 2016;95(5):1077. 27601522 10.4269/ajtmh.16-0383 PMC5094220

[71] Griffing SM, Mixson-Hayden T, Sridaran S, Alam MT, McCollum AM, Cabezas C, et al. South American *Plasmodium falciparum* after the malaria eradication era: clonal population expansion and survival of the fittest hybrids. PLoS ONE. 2011;6(9):e23486. 21949680 10.1371/journal.pone.0023486 PMC3174945

[72] Nkhoma SC, Nair S, Al-Saai S, Ashley E, McGready R, Phyo AP, et al. Population genetic correlates of declining transmission in a human pathogen. Mol Ecol. 2013;22(2):273–85. 23121253 10.1111/mec.12099 PMC3537863

