# Supplementary material for: Temporal changes in Plasmodium falciparum genetic diversity and multiplicity of infection across three areas of varying malaria transmission intensities in Uganda
Source: Trop Med Health. 2024 Dec 30;52:103. doi: 10.1186/s41182-024-00672-7 (PMC11684243; doi:10.1186/s41182-024-00672-7)
Supplement: Supplementary file 1 — Supplementary material 1. [file 41182_2024_672_MOESM1_ESM.doc]

**Temporal changes in *Plasmodium falciparum* genetic diversity and multiplicity of infection across three areas of varying malaria transmission intensities in Uganda**

**Table S1.** Microsatellite genotyping

**Microsatellite primer sequences used to amplify *Plasmodium falciparum*** isolates

| Primer  Name | Sequence | Tag | Length of product in 3d7 | Chromosome | Strand | Tandem repeats |
| --- | --- | --- | --- | --- | --- | --- |
| Neu1_TA1-F_HEX | CCGTCATAAGTGCAGAGC | 5HEX | 186bp | 6 | Minus | 3 |
| Neu1_TAI_R | TTTTATCTTCATCCCCAC | None |  |  | Plus |  |
| Neu1_TA1_F | CTACATGCCTAATGAGCA | None |  |  | Minus |  |
| NEU3_POLYA_F | AAAATATAGACGAACAGA | None | 151bp | 4 | Minus | 3 |
| NEU3_POLYA_R | ATCAGATAATTGTTGGTA | None |  |  | Plus |  |
| NEU3_POLYA_R_FAM | GAAATTATAACTCTACCA | 5 FAM |  |  | Plus |  |
| NEU7_PFPK2-F | CTTTCATCGATACTACGA | None | 171bp | 12 | Minus | 3 |
| NEU_PFPK2-R | CCTCAGACTGAAATGCAT | None |  |  | plus |  |
| NEU_PFPK2-R-HEX | AAAGAAGGAACAAGCAGA | 5HEX |  |  | Plus |  |
| NEU9_TA109_F | TAGGGAACATCATAAGGAT | None | 174bp | 6 | Plus | 3 |
| NEU9_TA109_R | CCTATACCAAACATGCTAAA | None |  |  | Minus |  |
| NEU9_TA109_F_FAM | GGTTAAATCAGGACAACAT | FAM |  |  | Plus |  |
| NEU12_2490-F | TTCTAAATAGATCCAAAG | None | 83bp | 10 | Plus | 3 |
| NEU12_2490-R | ATGATGTGCAGATGACGA | None |  |  | Minus |  |
| NEU12_2490_R_FAM | AGAATTATTGAATGCAC | 5FAM |  |  | Minus |  |
| 313_F_FAM | TCCCTTTTAAAATAGAAGAAA | 5FAM | 260bp | 2 | Plus | 2 |
| 313_R | GATTATATGAAAGGATACATG | None |  |  | Minus |  |
| 383_F_HEX | AATAGGAACAAATCATATTG | 5HEX | 173bp | 3 | Plus | 2 |
| 383_R | AGATATCCAGGTAATAAAAAG | NONE |  |  | minus |  |

**Master mix components and preparation**

| **Component** | **Stock concentration** | **Final concentration** | **Volume for one reaction (µL)** |
| --- | --- | --- | --- |
| PCR water | …….. | …… | 10.5 |
| Forward primer | 10 µM | 0.2 µM | 0.3 |
| Reverse primer | 10 µM | 0.2 µM | 0.3 |
| Buffer | 10X | 1X | 1.5 |
| dNTPs (2 mM), 0.75 MgCl_2_ (50mM), |  | 200 µM | 0.3 |
| Taq Polymerase | 5 U/µL | 1.5 mM | 0.1 |
| Template DNA |  |  | 2 |
| Final Volume |  |  | 15 µL |

**PCR conditions**

Microsatellites: Poly-α, TA1, TA109, PfPK2 and 2490

| **Stages** | **Steps** | **Primary round** | | | **Nested round** | | |
| --- | --- | --- | --- | --- | --- | --- | --- |
|  |  | **Temperature (^0^C)** | **Time** | **Cycles** | **Temperature (^0^C)** | **Time** | **Cycles** |
| Initial denaturation |  | 94 | 2 min | 1 | 94 | 2 min | 1 |
| 2. PCR | 1. Denaturation | 94 | 30 sec | 25 | 94 | 20 sec | 25 |
|  | 2. Annealing | 42  40 | 30 sec  30 sec |  | 45 | 20 sec |  |
|  | 3. Extension | 65 | 40 sec |  | 65 | 30 sec |  |
| 3. Final elongation & Hold | 1. Final elongation | 65 | 2 min | 1 | 65 | 2 min |  |
|  | 2. Hold | 4 | ∞ |  | 4 | ∞ |  |

**Microsatellites:** **C2M34–313, and C3M69–383**

| **Stages** | **Steps** | **Temperature (^0^C)** | **Time** | **Cycles** |
| --- | --- | --- | --- | --- |
| Initial denaturation |  | 94 | 2 min | 1 |
| 1 step. PCR | 1. Denaturation | 94 | 30 sec | 5 |
|  | 2. Annealing | 50 | 30 sec |  |
|  | 3. Extension | 60 | 30 sec |  |
| 2^nd^ step PCR | Denaturation | 94 | 30 sec | 40 |
|  | Annealing | 45 | 30 sec |  |
|  | Extension | 60 | 30 sec |  |
| 3. Final elongation & Hold | 1. Final elongation | 60 | 2 min | 1 |
|  | 2. Hold | 4 | ∞ |  |
