# Supplementary material for: Temporal changes in Plasmodium falciparum genetic diversity and multiplicity of infection across three areas of varying malaria transmission intensities in Uganda
Source: Trop Med Health. 2024 Dec 30;52:103. doi: 10.1186/s41182-024-00672-7 (PMC11684243; doi:10.1186/s41182-024-00672-7)
Supplement: Supplementary file 2 — Supplementary material 2. [file 41182_2024_672_MOESM2_ESM.doc]

**Temporal changes in *Plasmodium falciparum* genetic diversity and multiplicity of infection across three areas of varying malaria transmission intensities in Uganda**

**Table S2**. Genetic diversity of *P. falciparum* between 2011-2012 and 2015-2016 study periods across sites

| **Locus** | **Site** | **Isolates collected 2011-2012** | | | **Isolates collected 2015-2016** | | |
| --- | --- | --- | --- | --- | --- | --- | --- |
|  |  | N_a_ | N_e_ | H_e_ | N_a_ | N_e_ | H_e_ |
| 2490 | Jinja | 5 | 1.87 | 0.48 | 5 | 2.06 | 0.54 |
|  | Kanungu | 5 | 2.13 | 0.55 | 3 | 1.58 | 0.38 |
|  | Tororo | 4 | 1.68 | 0.42 | 4 | 2.15 | 0.55 |
| Poly α | Jinja | 11 | 8.01 | 0.90 | 10 | 6.38 | 0.88 |
|  | Kanungu | 12 | 9.23 | 0.93 | 12 | 7.92 | 0.90 |
|  | Tororo | 11 | 6.68 | 0.89 | 11 | 8.04 | 0.91 |
| C2M34–313 | Jinja | 14 | 9.09 | 0.92 | 14 | 11.26 | 0.95 |
|  | Kanungu | 15 | 12.76 | 0.96 | 14 | 8.50 | 0.91 |
|  | Tororo | 17 | 9.85 | 0.94 | 15 | 11.76 | 0.96 |
| TA1 | Jinja | 10 | 7.76 | 0.89 | 9 | 5.95 | 0.87 |
|  | Kanungu | 10 | 4.96 | 0.83 | 11 | 6.28 | 0.87 |
|  | Tororo | 10 | 5.79 | 0.88 | 11 | 6.87 | 0.89 |
| TA109 | Jinja | 6 | 3.30 | 0.71 | 6 | 4.69 | 0.72 |
|  | Kanungu | 5 | 4.07 | 0.78 | 7 | 4.78 | 0.85 |
|  | Tororo | 7 | 4.30 | 0.92 | 9 | 5.78 | 0.85 |
| C3M69–383 | Jinja | 9 | 4.13 | 0.78 | 6 | 2.11 | 0.55 |
|  | Kanungu | 7 | 3.13 | 0.71 | 7 | 3.35 | 0.52 |
|  | Tororo | 9 | 4.96 | 0.83 | 10 | 4.35 | 0.57 |
| PfPK2 | Jinja | 8 | 5.64 | 0.84 | 7 | 5.04 | 0.83 |
|  | Kanungu | 8 | 4.64 | 0.81 | 9 | 3.12 | 0.70 |
|  | Tororo | 7 | 5.45 | 0.85 | 6 | 5.06 | 0.83 |
| Overall (Mean ± SD) | Jinja | 9.0 | 5.68 | 0.79 | 8.14 | 5.36 | 0.76 |
|  | Kanungu | 8.86 | 5.85 | 0.80 | 9.00 | 5.08 | 0.73 |
|  | Tororo | 9.29 | 5.53 | 0.82 | 9.43 | 6.29 | 0.79 |
