# Supplementary material for: Temporal changes in Plasmodium falciparum genetic diversity and multiplicity of infection across three areas of varying malaria transmission intensities in Uganda
Source: Trop Med Health. 2024 Dec 30;52:103. doi: 10.1186/s41182-024-00672-7 (PMC11684243; doi:10.1186/s41182-024-00672-7)
Supplement: Supplementary file 3 — Supplementary material 3. [file 41182_2024_672_MOESM3_ESM.doc]

**Temporal changes in *Plasmodium falciparum* genetic diversity and multiplicity of infection across three areas of varying malaria transmission intensities in Uganda**

**Table S3.** *P. falciparum* percentage of polyclonal infection and mean MOI between 2011-2012 and 2015-2016 study periods sites

| **Locus** | **Site** | **Isolates collected 2011-2012** | | **Isolates collected 2015-2016** | |
| --- | --- | --- | --- | --- | --- |
|  |  | Percentage polyclonal | Mean MOI | Percentage polyclonal | Mean MOI |
| 2490 | Jinja | 36.8 | 1.4 | 40.0 | 1.5 |
|  | Kanungu | 34.8 | 1.1 | 36.5 | 1.3 |
|  | Tororo | 45.8 | 1.5 | 38.7 | 1.4 |
| Poly-α | Jinja | 47.4 | 1.9 | 40.0 | 1.8 |
|  | Kanungu | 59.3 | 1.9 | 50.0 | 2.0 |
|  | Tororo | 50.0 | 2.0 | 66.7 | 2.3 |
| C2M34–313 | Jinja | 70.3 | 2.1 | 81.8 | 1.8 |
|  | Kanungu | 34.0 | 1.3 | 42.4 | 1.4 |
|  | Tororo | 50.0 | 1.7 | 54.2 | 1.6 |
| TA1 | Jinja | 76.3 | 2.2 | 54.2 | 1.9 |
|  | Kanungu | 81.5 | 2.4 | 52.4 | 2.3 |
|  | Tororo | 72.2 | 2.1 | 74.6 | 2.2 |
| TA109 | Jinja | 52.6 | 1.9 | 44.0 | 2.0 |
|  | Kanungu | 45.9 | 1.5 | 45.5 | 1.5 |
|  | Tororo | 66.7 | 2.4 | 71.0 | 2.2 |
| C3M69–383 | Jinja | 84.4 | 2.6 | 81.0 | 2.4 |
|  | Kanungu | 70.4 | 2.0 | 73.5 | 1.7 |
|  | Tororo | 82.6 | 2.8 | 86.2 | 2.8 |
| PfPK2 | Jinja | 57.9 | 2.0 | 56.0 | 2.0 |
|  | Kanungu | 66.7 | 1.9 | 69.4 | 2.0 |
|  | Tororo | 95.7 | 2.8 | 70.0 | 1.7 |
| Overall | Jinja | 60.8 | 2.0 | 56.7 | 1.9 |
|  | Kanungu | 56.1 | 1.7 | 52.8 | 1.7 |
|  | Tororo | 66.1 | 2.2 | 65.9 | 2.0 |
